# Homozygous mutation of the LRRK2 ROC domain as a novel genetic model of parkinsonism

**DOI:** 10.1186/s12929-022-00844-9

**Published:** 2022-08-14

**Authors:** Meng-Ling Chen, Ruey-Meei Wu

**Affiliations:** 1 Department of Neurology, National Taiwan University Hospital, College of Medicine, National Taiwan University, No. 7, Chung-Shan South Road, Taipei, 10002 Taiwan; 2 Department of Life Science, College of Life Science, National Taiwan University, No. 1, Sec. 4, Roosevelt Road, Taipei, 10617 Taiwan

**Keywords:** Anxiety, Fission, Gait, GTPase activity, Homozygous, *LRRK2*, Parkinsonism, PET, R1441G

## Abstract

**Background:**

Parkinson’s disease (PD) is one of the most important neurodegenerative disorders in elderly people. Mutations in the leucine-rich repeat kinase 2 (*LRRK2*) gene are found in a large proportion of the patients with sporadic and familial PD. Mutations can occur at different locations in the *LRRK2*. Patients with LRRK2 ROC-COR mutations face an increased risk of typical motor symptoms of PD, along with cognitive decline. An animal model with a monogenic *LRRK2* gene mutation is a suitable model for exploring the pathophysiology of PD and identifying potential drug therapies. However, the effect of homozygous (HOM) *LRRK2* in PD pathophysiology is unclear.

**Methods:**

We established human *LRRK2* (*hLRRK2*) R1441G HOM transgenic (Tg) mice to explore the phenotype and pathological features that are associated with *hLRRK2* R1441G Tg mouse models and discuss the potential clinical relevance. The open field test (OFT) was performed to examine motor and nonmotor behaviors. A CatWalk analysis system was used to study gait function. [^18^F]FDOPA PET was used to investigate functional changes in the nigrostriatal pathway in vivo. Transmission electron microscopy was used to examine the morphological changes in mitochondria and lysosomes in the substantia nigra.

**Results:**

The R1441G HOM Tg mice demonstrated gait disturbance and exhibited less anxiety-related behavior and exploratory behavior than mice with *hLRRK2* at 12 months old. Additionally, [^18^F]FDOPA PET showed a reduction in FDOPA uptake in the striatum of the HOM Tg mice. Notably, there was significant lysosome and autophagosome accumulation in the cytoplasm of dopaminergic neurons in R1441G hemizygous (HEM) and HOM mice. Moreover, it was observed using transmission electron microscopy (TEM) that the mitochondria of R1441G Tg mice were smaller than those of *hLRRK2* mice.

**Conclusion:**

This animal provides a novel HOM *hLRRK2* R1441G Tg mouse model that reproduces some phenotype of Parkinsonism in terms of both motor and behavioral dysfunction. There is an increased level of mitochondrial fission and no change in the fusion process in the group of HOM *hLRRK2* R1441G Tg mouse. This mutant animal model of PD might be used to study the mechanisms of mitochondrial dysfunction and explore potential new drug targets.

**Supplementary Information:**

The online version contains supplementary material available at 10.1186/s12929-022-00844-9.

## Background

Mutations in leucine-rich repeat kinase 2 (*LRRK2*) are the one of the most common genetic causes of Parkinson’s disease (PD). Mutations can occur at various locations in *LRRK2*. To date, eight mutations in *LRRK2*, including N1437H, R1441 G ⁄H⁄ C, Y1699C, I2012T, G2019S, and I2020T, have been shown to be associated with PD [15, 30, 34]. G2019S is the most common mutation, accounting for 6–42% in familial PD cases and 3–34% in sporadic PD cases in the North African, Ashkenazi Jewish, European and North American populations [6, 12, 19, 24, 27, 41]. However, this mutation is rare in Asian populations. The second most common mutation consists of the R1441 “hotspot” amino acid codon residues of glycine (G), histidine (H), and cysteine (C) individually. R1441G is most prevalent in the Basque region of Spain (16.4–46% of familial PD cases and 1.7–4% of sporadic PD cases) [17, 21, 50]. Although most PD patients with *LRRK2* mutations are heterozygous missense mutations with an autosomal dominant inheritance pattern, there is a large population of patients carrying homozygous (HOM) mutations in *LRRK2* R1441G and G2019S worldwide [2, 25, 26, 58]. Patients with mutations in the *LRRK2* gene show the phenotypic features of sporadic PD, including not only motor symptoms but also nonmotor symptoms such as depression, and cognitive impairment. [28, 34, 53].

Mammalian LRRK2 is a 2527-residue protein, with a catalytic core domain, a kinase domain and a number of putative protein–protein interaction domains. The catalytic core domain consists of a Ras GTPase-like domain termed ROC (Ras of complex protein) followed by a COR (carboxy-terminal of Ras) domain immediately before the kinase domain [9]. The regulation of LRRK2 kinase activity depends on the ROC domain of the dimer, and dimerization may depend on the COR domain as a molecular hinge. Various *LRRK2* mutation in vitro and in vivo assays have been established and demonstrated that mutations in *LRRK2* could be involved in the pathogenesis of PD through the autophagic–lysosomal pathway, intracellular trafficking, mitochondrial dysfunction, and the ubiquitin–proteasome system [45]. Inhibitors of LRRK2 kinase have been introduced in clinical trials for symptomatic treatment of sporadic PD and monogenic PD patients with *LRRK2* mutations [55].

Many animal models of *LRRK2* heterozygous gene mutation have been established in the last decade [59, 62]. However, most mouse models do not show visible PD symptoms under normal conditions but are more susceptible to external stress [35, 36, 56, 64]. Only a few studies have focused on the mutation of the ROC-COR domain and investigated the underlying molecular pathways of neurodegeneration.

Herein, we generated HOM human *LRRK2* (*hLRRK2*) R1441G mice to evaluate motor function, gait analysis, anxiety and metabolic neuroimaging (small animal PET/CT imaging of [^18^F]FDOPA). Moreover, we further measured the GTPase activity and total protein expression of LRRK2 with and without phosphorylation and examined the ultrastructural changes in mitochondria and lysosomes in the midbrain. Next, we investigated the pathophysiology of the *hLRRK2* R1441G mutation on the regulation of mitochondrial fission/fusion dynamics and the autophagy pathway in this animal model.

## Methods

### Mice

All animal experimental procedures were approved by the Committee on Animal Research of National Taiwan University and carried out in accordance with the guidelines of the Committee. All mice were bred in the Laboratory Animal Center, National Taiwan University College of Medicine. All animals were housed in groups at 20–25 °C with 60% relative humidity, a 12/12 h light/dark cycle, and free access to food and water. Hemizygous (HEM) wild-type (WT) *hLRRK2* mice (FVB/N-Tg (*LRRK2*)1Cjli/J, no. 009610) [33], and HEM *hLRRK2* R1441G transgenic (Tg) mice (FVB/N-Tg (LRRK2*R1441G)135Cjli/J, no. 009604) [33] were purchased from the Jackson Laboratory and bred on an FVB/N background (Jackson stock number. 001800). HOM *hLRRK2* R1441G Tg mice were generated by crossing HEM *hLRRK2* R1441G Tg mice with HEM *hLRRK2* R1441G Tg mice. The HEM wild type *hLRRK2* was used as an ideal control. HEM and HOM *hLRRK2* R1441G Tg mice were used as comparison groups. Genomic DNA was obtained from mouse ear biopsies and amplified by polymerase chain reaction (PCR) and quantitative PCR (qPCR) using sequencing primers designed as described below. PD is found more frequently in men than in women, and only male mice were used for experiments in this study.

### Genotyping HOM

Genomic DNA was extracted from 5 mm ear punch biopsies by lysing the tissue in 10 mg/ml proteinase K (in 50 mM Tris pH 8.0, 2 mM NaCl, 10 mM EDTA, 1% SDS) at 65 °C overnight followed by salt extraction and ethanol precipitation. The mice were genotyped using genomic DNA from the ear biopsy tissue was used to identify the mouse genotype. The following primers were used for PCR amplification of *hLRRK2*: 5ʹ-TGATTCTCGTTGGCACACAT-3ʹ and 5ʹ-GCCAAAGCATCAGATTCCTC-3ʹ. PCR was conducted in a 20 μl reaction volume containing approximately 1 μl genomic DNA, 0.5 μl of each 100 μM primer, and 10 μl 2× Master Mix (Promega). The thermocycler conditions were set to 94 °C for 2 min 30 s, then 35 cycles of 94 °C for 30 s, 68 °C for 1 min and 72 °C for 1 min, followed by 72 °C for 2 min. To identify the genotype of the mouse model as either HOM or HEM for *hLRRK2*, the other sets of primers were used for real-time qPCR: For the *hLRRK2* gene, the primer and probe sequences were as follows: forward primer 5′-GCATTAGAGATGTTATCCCTGGAA-3′, reverse primer 5′-GTACTGACCTTGGTCATCTGGATA-3′, and probe 5′-FAM-ATGGATTCAGTGCTTCACACACTGCA-BHQ1-3′. FAM is a fluorescent reporter and BHQ1 is a quencher fluorophore. The apolipoprotein B gene (apoB) was used as an internal control to normalize variations in the amount of input DNA. The product size was 73 bp. The primer and probe sequences were as follows: forward primer 5′-CACGTGGGCTCCAGCATT-3′, reverse primer 5′-TCACCAGTCATTTCTGCCTTTG-3′, and probe 5′-Cy5-CCAATGGTCGGGCACTGCTCAA-BHQ2-3′. Cy5 was a second fluorescent reporter. qPCR was conducted in a 20 μl reaction volume containing approximately 50 ng genomic DNA, 0.8 μl of each 10 μM primer and 0.3 μl of each 10 μM probe, and 10 μl 2× qPCR Master Mix (Promega). The thermocycler conditions were set to 95 °C for 2 min, followed by 40 cycles of 95 °C for 15 s, and 60 °C for 1 min.

### Behavior assay

All the behavioral tests on all mice were performed at the same time of day.

### Spontaneous locomotor activity

All procedures were performed in the dark as an adaptation to typical mouse behavior. Mice were placed in a corner of an open-field apparatus (16 × 16 × 15 inches, San Diego Instruments, San Diego, CA, USA) consisting of a Plexiglas cages white floors and translucent walls. Locomotor activity was monitored using a 16 × 16 array of photobeams (beam interval, 1 inch). The sampling rate was once per second. The patterns of beam breaks were computed (Photobeam Activity System—Open Field, San Diego Instruments, San Diego, CA, USA) to obtain parameters of locomotor activity. Data were collected for 30 min and each time in a 10-min period over three consecutive periods.

### Spontaneous gait analysis

All procedures were performed in the dark (except for light emitted from the nearby computer screen) to enhance the contrast of the paw print images. The CatWalk system (Noldus Information Technology, Wageningen, Netherlands) was used to analyze the gait of unforced moving mice. CatWalk includes a hardware system with a glass walkway plate, illuminated with green light that is reflected within the glass; at points of contact, the light is reflected toward a high-speed video camera. CatWalk software 10.0 was used for quantitative assessment of animal footprints. A successful run was defined as a complete run along the tracks without any interruption or hesitation. Mice that failed the CatWalk training were excluded from the study. The average number of 5 replicate crossings made by each mouse was recorded. Mice were subjected to computer-assisted CatWalk testing every month for 1 year.

### [^18^F]FDOPA micro PET

[^18^F]FDOPA was prepared and synthesized at the Department of Nuclear Medicine at National Taiwan University Hospital. All PET scans were acquired on an Argus PET/CT (SEDECAL) with a spatial resolution of 1.1 mm, a transaxial field of view (FOV) of 68 cm and an axial FOV of 4.7 cm. Animals were anesthetized and maintained with a mixture of 1.5% isoflurane and nitrous oxide:oxygen (7:3). Static scans were acquired 30 min after a single bolus injection of [^18^F]FDOPA (12.9 ± 1.47 MBq in 0.1 ml saline) via the tail vein in 3D mode for 60 min. Briefly, images were reconstructed using the 2D ordered-subset expectation maximization (OSEM) algorithm with radians and scatter corrections and without attenuation. There were a total of 61 slices of reconstructed images, each with a matrix size of 175 × 175. The correction and images were analyzed in PMOD software (version 3.2, PMOD Technologies), and the volumes of interest (VOIs) were drawn over the right and left striatum and cerebellum (CB) in irregular shapes. To ensure proper VOI placement, PET images were coregistered with a mouse MRI template. The quantitative analysis of [^18^F]FDOPA uptake in brain regions was first shown as a standardized uptake value (SUV) by the formula: SUV measured tissue activity [Bq/ml]/(injected dose [Bq]/body weight [g]). The SUV ratio (SUVR) of [^18^F]FDOPA was calculated by (the sum of right and left striatum uptake − cerebellar uptake)/cerebellar uptake with cerebellum as the reference region.

### Determination of GTPase (guanosine triphosphatase) levels

GTPase activity in tissue sample lysates was determined using an ATPase/GTPase ELIPA Biochem Kit (Cytoskeleton, Denver, CO, USA) that measures the amount of inorganic phosphate (Pi) generated during hydrolysis on a real-time basis, according to the manufacturer’s instructions.

### Transmission electron microscopy (TEM)

For TEM analysis, mice were deeply anesthetized by intraperitoneal injection of sodium pentobarbital and transcardially perfused with ice-cold 0.9% saline and 4% paraformaldehyde (PFA) in 0.1M phosphate buffered saline (PBS, pH 7.4). The substantia nigra pars compacta (SNc) of mice were then cut into 1 mm^3^ squares and postfixed in Trump’s solution (4% formaldehyde + 0.1% glutaraldehyde in 0.1M phosphate buffer) for 1 h at room temperature. Subsequently, the samples were kept in the 2% glutaraldehyde in 0.1M Na-cacodylate buffer (pH 7.4) overnight. Tissue was fixed in 1% osmium tetroxide and 1% aqueous uranyl acetate, dehydrated in a graded series of ethanol, and embedded in Embed 812/Araldite (EMS, Hatfield, PA). Thin sections (0.1 μm) were collected on copper grids, poststained with lead citrate and viewed at 80 kV with a JEOL 1400 transmission electron microscope (JEOL USA, Peabody, MA) or TEM H-600 (HITACHI, Tokyo, Japan).

### Tissue dissection

Mice were euthanized with CO_2_, followed by decapitation. The tissues were quickly isolated and cooled in ice-cold saline. The brains were placed on an ice-cold brain matrix (Stoelting) for further dissection of the indicated brain areas such as the SNc and striatum. For western blotting (WB), the tissues were immediately frozen and stored at − 80 °C until assayed.

### Fractionation of mitochondria

Briefly, fresh brains were obtained within 1 h of sacrifice and kept on ice. A Mitochondria Isolation Kit (Sigma-Aldrich) was used for mitochondrial fractionation, and the procedures followed the manufacturer’s instructions. The purified mitochondria were stored at – 70 °C or subjected to the next analysis.

### Protein extraction

Total proteins were prepared from whole brain, SNc, and striatum tissues for western blotting analysis. Frozen tissue samples were homogenized with a microhomogenizer in ice-cold CelLytic™ MT Cell Lysis Reagent (Sigma-Aldrich) containing 1× Protease Inhibitor Cocktail (Sigma-Aldrich) and 1× PhosSTOP™ (Roche Diagnostics Ltd.). After tissue disruption, homogenates were centrifuged at 12,000×*g* for 10 min to pellet the tissue debris. The supernatants were transferred to a clean test tube.

### Western blotting

The protein concentration of brain capillary membrane samples was determined using a Bradford protein assay. Normalized brain capillary membrane samples were separated and transferred using the NuPAGE electrophoresis and blotting system (Invitrogen). After protein transfer, the blotting membranes were incubated overnight with primary antibody (Fis1, Genetex; Drp1 (D6C7), Cell Signaling; LC3B, Genetex; LRRK2 [MJFF (c41-2)], Abcam; SQSTM1/p62 [EPR4844], Abcam). After antibody incubation, the membrane was washed and incubated with the corresponding HRP-conjugated secondary antibody (1:5,000; Genetex). Proteins were detected using Immobilon Western Chemiluminescent HRP Substrate (Millipore), and protein bands were visualized with a VisionWorks^®^LS Analysis Software (UVP Inc.) and analyzed with ImageJ as an arbitrary optical density unit.

### Statistical analysis

We performed all statistical analyses in GraphPad Prism. We analyzed the effect of one variable on more than two groups using a one-way ANOVA and pairwise t-tests with a Bonferroni correction, Dunn’s test, or Holm–Sidak post hoc analysis. We analyzed the effect of two variables using a two-way ANOVA and pairwise t tests with a Bonferroni correction, Dunn’s test, or Holm–Sidak post hoc analysis. The statistical significance threshold was *p* < 0.05 for all tests. *< 0.05, **< 0.01, ***< 0.001, ****< 0.0001.

## Results

### Poor growth and abnormal GTPase and Ser935 expression

**Fig. 1:**
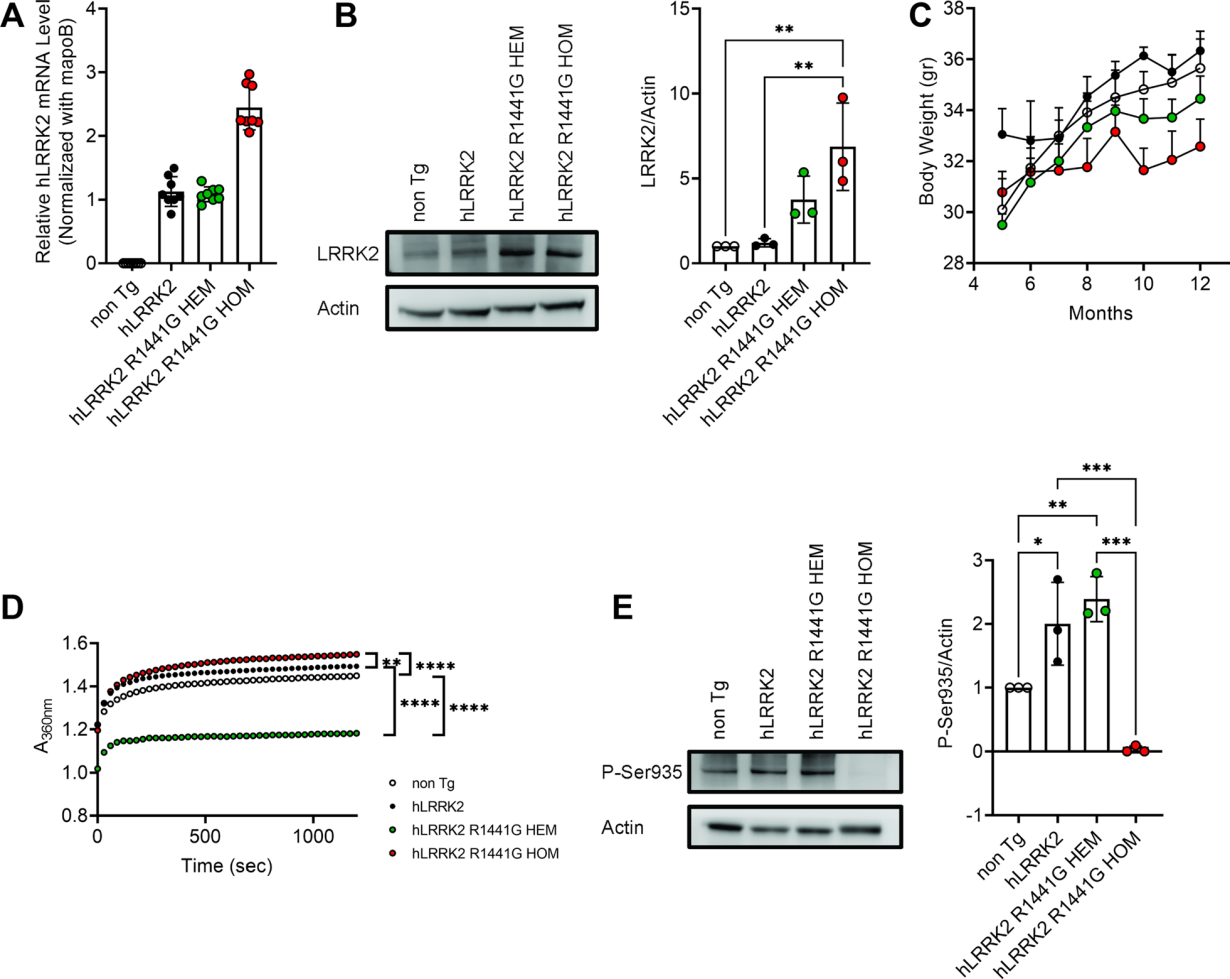
Detection of *hLRRK2* and *hLRRK2* R1441G expression and GTPase activity in Tg mice. **A** Real-time PCR assays of *hLRRK2* and *hLRRK2* R1441G mRNA expression in the brains of Tg mice. Data represent relative mRNA levels (mean ± SEM, n = 8) normalized to mouse apoB and are expressed in arbitrary units. **B** LRRK2 protein expression in the brains of non-Tg and Tg mice (n = 3). **C** *hLRRK2* R1441G HEM and HOM mice lost less weight than non-Tg mice. Data are presented as the mean ± SEM (n = 6). **D** GTPase activity was measured using an enzyme-linked inorganic phosphate assay (ELIPA; n = 3). **E** Disruption of LRRK2 Ser935 phosphorylation in the brains of *hLRRK2*, R1441G HOM mice. Total lysates from the brains of 12-month-old *hLRRK2* R1441G HOM mice were analyzed by western blotting for phosphorylation of LRRK2 at Ser935; actin was used as a loading control. *Significant effect, p < 0.05; **significant effect, p < 0.01; ***significant effect, p < 0.005; ****significant effect, p < 0.001

Earlier studies reported that HEM *hLRRK2* R1441G Tg mice induce age-dependent, levodopa-responsive slowness of movement associated with diminished dopamine release and axonal pathology of nigrostriatal dopaminergic projection [33]. However, these phenotypes could not always be replicated in independent studies. Compared to the non-Tg mice, previous studies have reported that HEM *hLRRK2* R1441G mice significantly decreased motor function and non-motor behavior at 15 months old [13] or 20 months old [7]. Therefore, we generated HOM *LRRK2* R1441G mice. We used real-time PCR analyses to identify *hLRRK2* R1441G HOM mice. As shown in Fig. 1A, the expression of exogenously introduced *hLRRK2* mRNA was elevated in HOM mice. The mRNA of HOM *hLRRK2* R1441G Tg mice is doubled when it was compared to the *hLRRK2* and HEM *hLRRK2* R1441G Tg mice. The immunoblots showed a significant difference in protein expression between non-Tg or *hLRRK2* mice and HOM *hLRRK2* R1441G mice in the brain at 3 months of age (Fig. 1B). When food and water were available ad libitum, both R144G HEM and HOM mice grew slower (Fig. 1C).

R1441G mutation suppresses GTPase activity and promotes GTP binding which in turn mediates a three to fourfold increase in LRRK2 kinase activity [49, 51]. We next collected SNc from 12-month-old mice to assess the effect of R1441G mutation on the expression of GTP hydrolysis. As shown in Fig. 1D, compared with that in *hLRRK2* mice, GTPase activity was significantly reduced in HEM *hLRRK2* R1441G mice, whereas significantly increased GTPase activity was found in HOM *hLRRK2* R1441G mice. Ser935, located prior to the leucine-rich repeat domain on the LRRK2 enzyme, is the most common site at which phosphorylation is measured [14]. We decided to investigate Ser935 phosphorylation to assess the LRRK2 phosphorylation. The data revealed that Ser935 phosphorylation was abolished in R1441G HOM mice (Fig. 1E).

### HOM mice have an abnormal motor and nonmotor behaviors

**Fig. 2:**
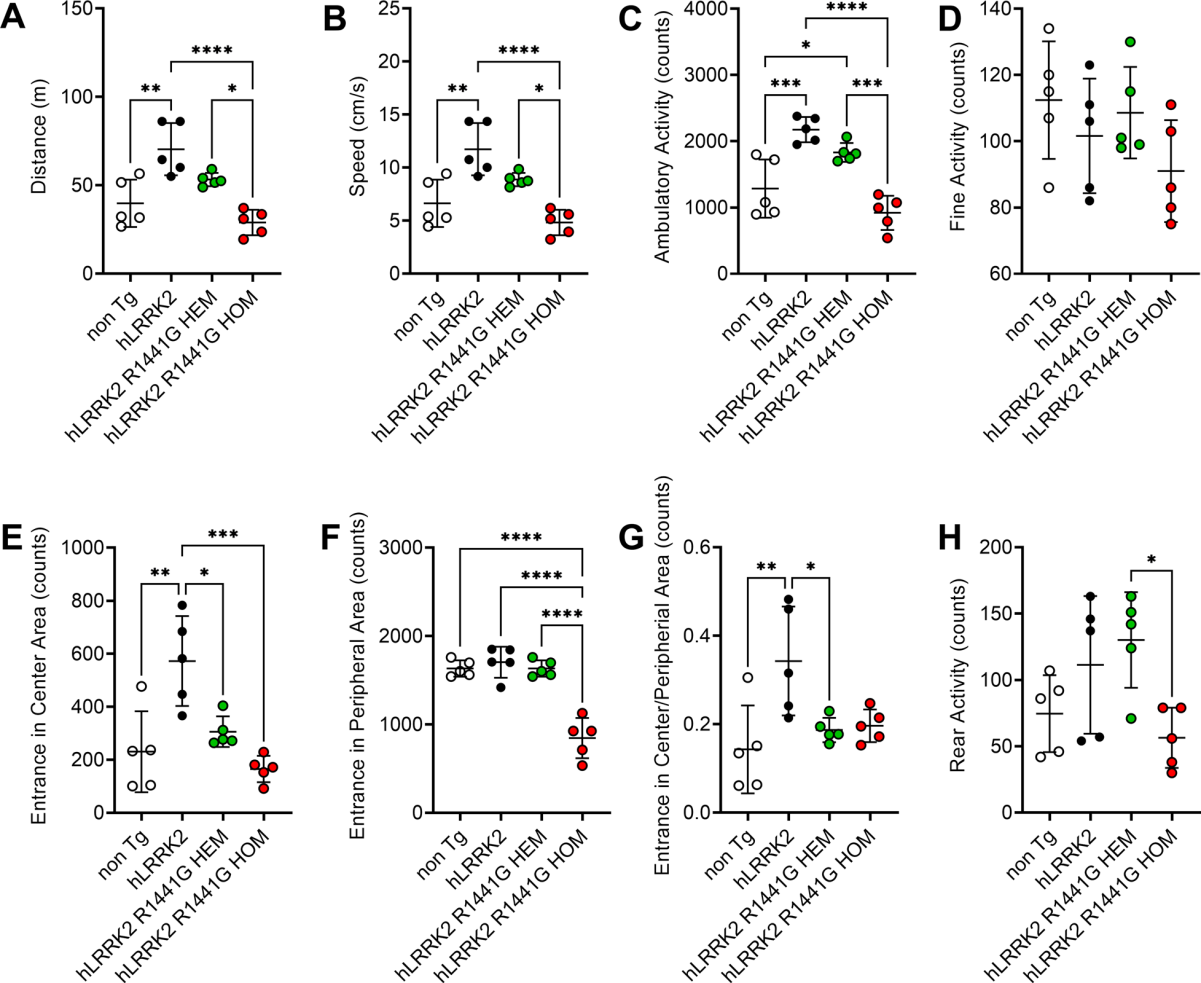
Locomotor activity in the open-field test was significantly reduced in *hLRRK2*2 R1441G HOM mice. Data are presented as the mean ± SD (n = 5). *Significant effect, p < 0.05; **significant effect, p < 0.01; ***significant effect, p < 0.005; ****significant effect, p < 0.001

**Fig. 3:**
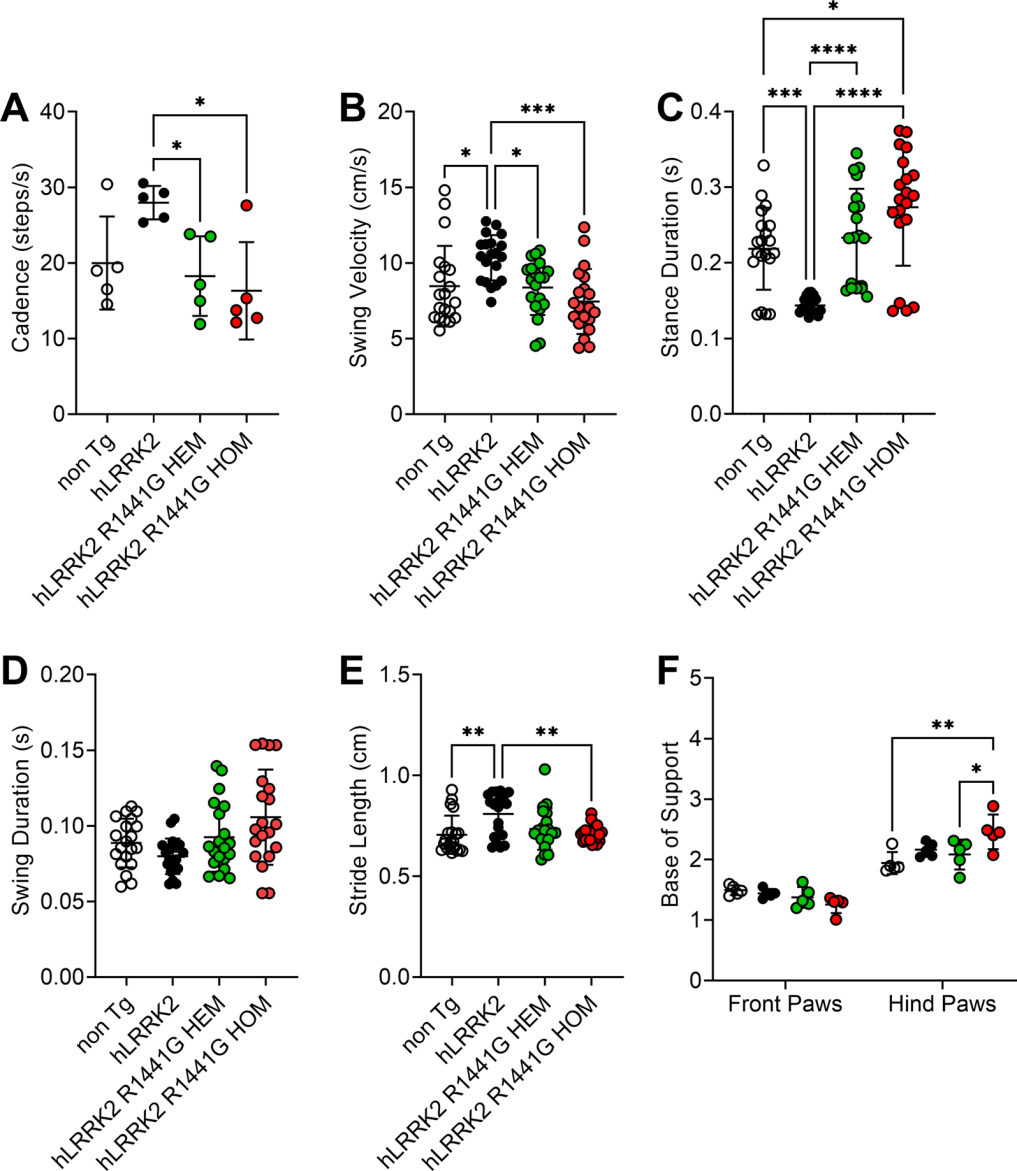
Several parameters of measured by the CatWalk system were affected in various ways in four mice. The swing velocity, cadence, and stride length were decreased in *hLRRK2* R1441G HOM mice. In contrast, the stance duration and the pressure on the hind-paws BOS increased in *hLRRK2* R1441G HOM mice. Data are presented as the mean ± SD (n = 5). *Significant effect, p < 0.05; **significant effect, p < 0.01; ***significant effect, p < 0.005; ****significant effect, p < 0.001

We then analyzed age-matched non-transgenic (non-Tg), *hLRRK2*, R1441G HEM and HOM male mice at 9, and 12 months in a battery of behavioral tests, including spontaneous locomotor activity levels (Fig. 2A–D), anxiety-related emotional (Fig. 2E–G) and exploratory behaviors (Fig. 2H), and gait analysis (Fig. 3).

First, the quantity of locomotion as assessed in the open field test the (OFT) was affected in HOM group, with decreased distance traveled (Fig. 2A, n = 5 per group) and average speed (Fig. 2B, n = 5 per group). When the Tg mice were placed in an open field, the ambulatory activity of 12-month-old R1441G HOM mice decreased significantly compared to 12-month-old *hLRRK2* and R1441G HEM mice (Fig. 2C, n = 5 per group).

To investigate whether HEM and HOM *hLRRK2* R1441G mice display nonmotor behaviors, we studied anxiety-related emotional and exploratory behaviors in freely moving HEM and HOM *hLRRK2* R1441G mice, *hLRRK2* mice and non-Tg mice using the OFT. The OFT is also widely used to assess the emotionality in rodents [48]. Compared to the non-Tg and *hLRRK2* mice, we found that the HOM group also significantly decreased the time spent in the peripheral (Fig. 2F, n = 5 per group) and central (Fig. 2E, n = 5 per group) zones of the maze statistically analyzed. Rearing is a common measure of activity and exploratory behavior used in the OFT. Moreover, R1441G HOM mice exhibited less exploratory rearing behavior in the dark phase than R1441G HEM mice at 12 months old (Fig. 2G, n = 5 per group). However, contrary to HOM *hLRRK2* R1441G mice, 12-month-old HEM *hLRRK2* R1441G mice had slightly increased rearing activity compared to the non-Tg mice. The data were consistent with a previous study [33].

Gait impairments are the most commonly observed clinical manifestation in PD patients [38]. To investigate whether HEM and HOM *hLRRK2* R1441G mice display the gait impairments, we compare 12-month-old heterozygote and homozygote *hLRRK2* R1441G, *hLRRK2* and non-Tg mice in the CatWalk system. The CatWalk system captures a substantial number of gait parameters, both dynamic and static. A significant difference was noted between the *hLRRK2* group and the HEM and HOM groups (Fig. 3, n = 5 per group). The gait activity as assessed in the CatWalk system with swing duration did not significantly change in all group (Fig. 3A, n = 5 per group). In contrast to the swing duration, swing velocity (cm/s) was shorter in the *hLRRK2* HOM group (Fig. 3B, n = 5). The stance duration is the time during which the paw is in contact with the glass plate. As shown in Fig. 3C, 12-month-old R1441G HOM mice showed significantly longer stance duration than 12-month-old non-Tg (n = 5) and *hLRRK2* mice (n = 5).

In contrast, Fig. 3D shows that cadence was shorter in R1441G HOM mice than in *hLRRK2* mice (Mean Diff = 11.66, *p* < 0.05, n = 5 per group). The stride length displays the distance between successive placements of the same paw. For stride length, HOM mice had a significantly shorter stride length than *hLRRK2* mice (Fig. 3E, Mean Diff = 0.1005, *p* < 0.01, n = 5 per group). For the base of support (BOS) of the hind limbs (Fig. 3F), it was noted that the HOM group placed significantly more units of pressure on the hind paws than the non-Tg groups (Fig. 3F, n = 5) and HEM mice (Fig. 3E, n = 5). Altogether, these results suggest that R1441G HOM mice exhibited a change in the quality of gait and decreased locomotor activity.

### PET assessment of dopamine depletion in the striatum

**Fig. 4:**
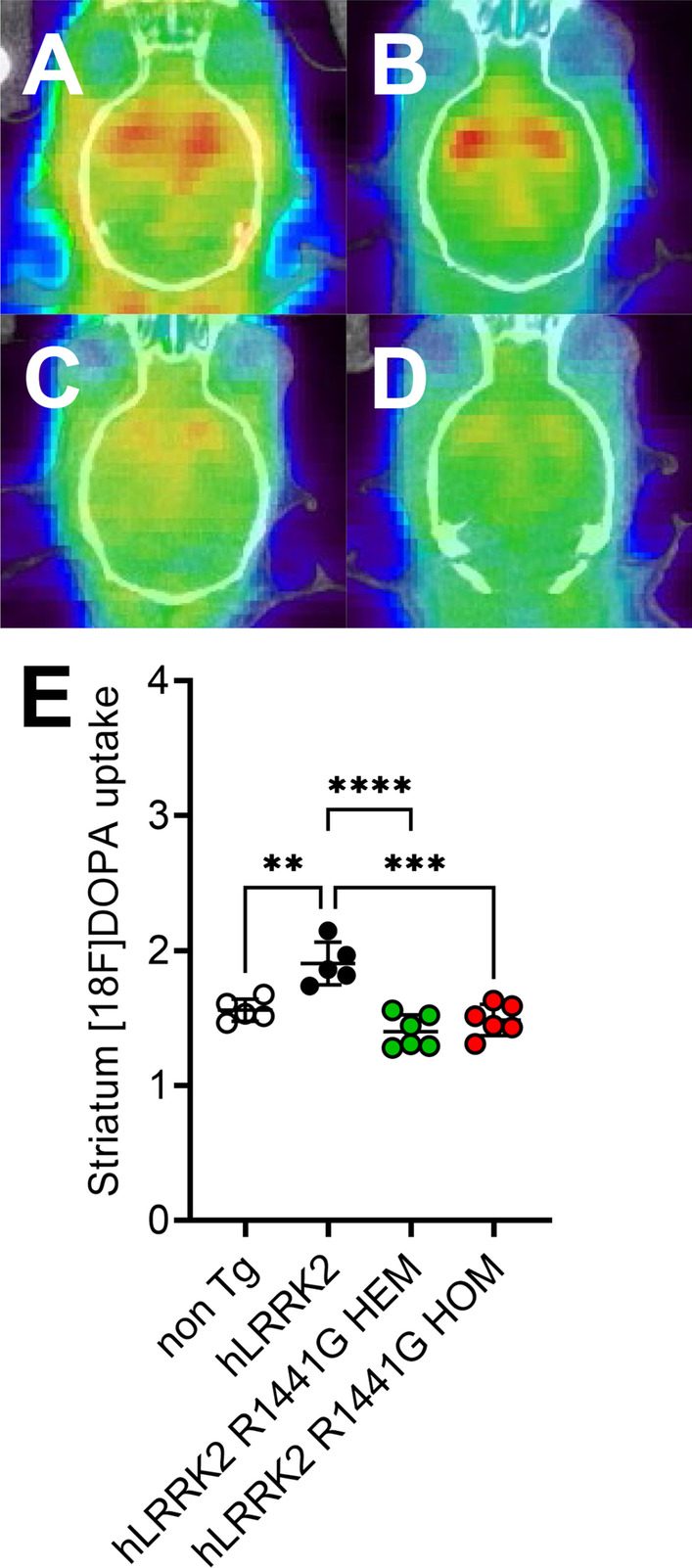
Comparison of [18F]FDOPA images from four groups of mice, showing significantly decreased uptake of the ligand among Tg mice: **A** coregistered coronal [^18^F]FDOPA images of the non-Tg mouse striatum; **B** coregistered coronal [^18^F]FDOPA images of the *hLRRK2* mouse striatum; **C** coregistered coronal [^18^F]FDOPA images of the *hLRRK2* R1441G HEM mouse striatum; **D** coregistered coronal [^18^F]FDOPA images of the *hLRRK2* R1441G HOM mouse striatum; **E** average [^18^F]FDOPA uptake in the region of interest (striatum) in various groups. The uptake values are **A**, **B**, **C**, and **D** for Group 1,2,3, and 4, respectively Data are presented as the mean ± SD (n = 5–6). *Significant effect, p < 0.05; **Significant effect, p < 0.01; ***Significant effect, p < 0.005; ****Significant effect, p < 0.001

[^18^F]FDOPA studies are the most widely and routinely used PET tracer for studying striatal changes in PD patients. When the depletion of dopamine in the striatum was measured by using PET imaging, all mice exhibited [^18^F]FDOPA uptake in the striatum (Fig. 4A–D). Figure 4A–D shows coregistered [^18^F]FDOPA PET images with computed tomography (CT), showing coronal brain slices of representative animals from the non-Tg (Fig. 4A), *hLRRK2* (Fig. 4B), *hLRRK2* R1441G HEM (Fig. 4C) and *hLRRK2* R1441G HOM (Fig. 4D) groups. Brain uptake of [^18^F]FDOPA in both non-Tg, and *hLRRK2* groups followed a similar pattern in the striatum. However, the image signals were lower in the *hLRRK2* R1441G HEM (Fig. 4C) and *hLRRK2* R1441G HOM (Fig. 4D) groups. As Fig. 4E shows, the average SUV for [^18^F]FDOPA in *hLRRK2* R1441G HEM and HOM mice was significantly lower than that in *hLRRK2* mice (n = 5–6 per group).

### Mutant-*hLRRK2* altered function and mitochondrial morphology in the brain

**Fig. 5:**
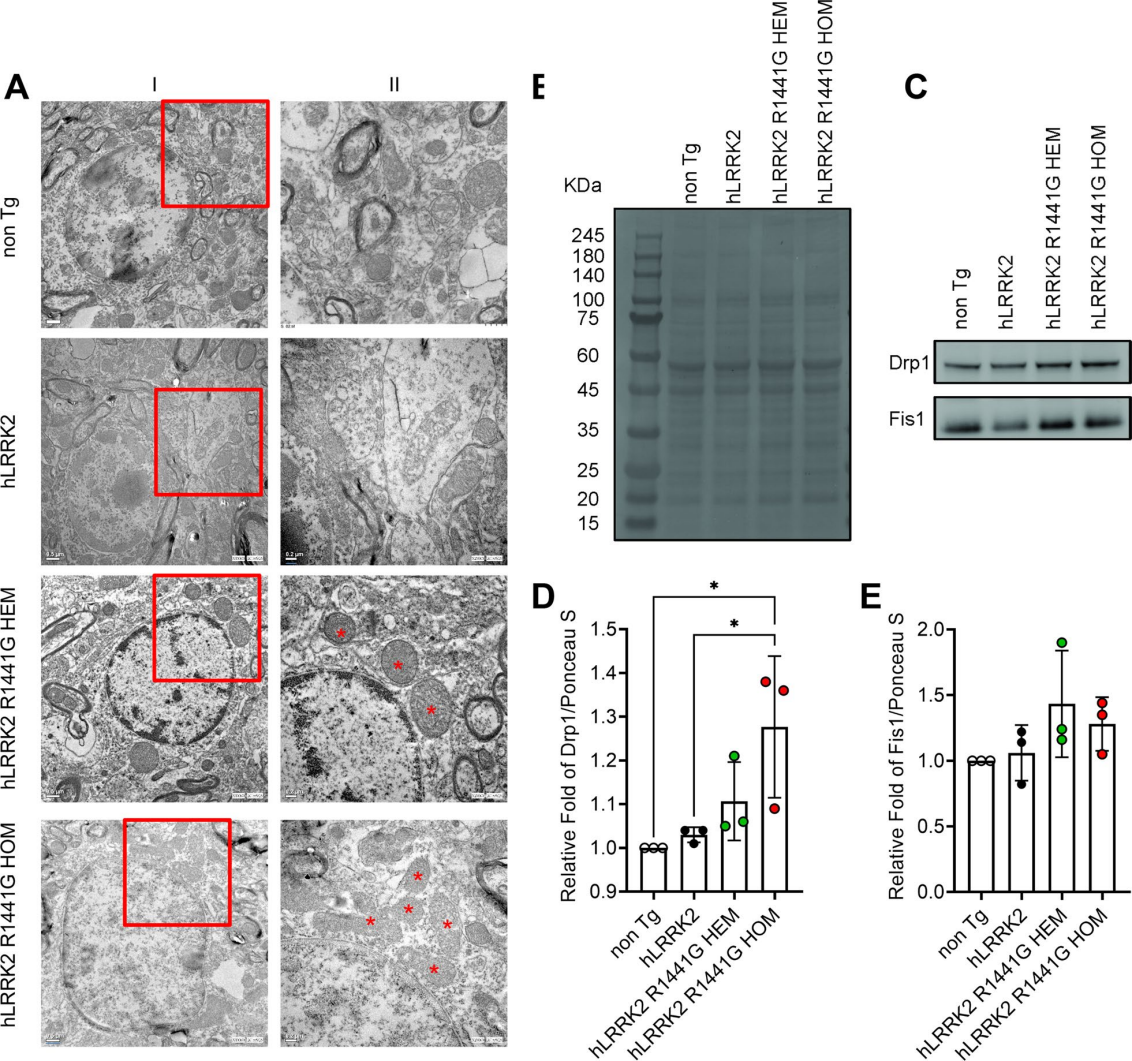
Effect of SN in *hLRRK2*, R1441G HEM and HOM mice SNc region. **A** Ultrastructural analysis of SN in *hLRRK2*, R1441G HEM and HOM mice SNc region. Non-Tg and *hLRRK2* represent a healthy mitochondrion; HEM and HOM represent a swollen mitochondrion. Selected regions in different magnification images (I, 10,000×; II, 20,000×). Asterisks indicate shrinkage and matrix condensation. HEM, R1441G HEM; HOM, R1441G HOM. **B** Western blot analysis of mitochondrial fission proteins in the mitochondrial fraction of the whole brain. Equal loading of the gel is demonstrated with Ponceau S staining for mitochondrial protein fragmentation performed on the blot before immunostaining. **B** Representative western blot for Drp1 and Fis1 expression. Densitometry of the Drp1 (**C**) and Fis1 (**D**) blots is shown as the fold increase (HEM or HOM *hLRRK2*). The figure shows a representative (of two) experiment. Values are means ± SD. *Significant effect, p < 0.05; **significant effect, p < 0.01; ***significant effect, p < 0.005; ****significant effect, p < 0.001

To determine if the *hLRRK2* R1441G HEM and HOM mutation can generate the effect of mitochondrial morphology, TEM images were collected from the SN of *hLRRK2*, R1441G HEM and HOM mice at 12 months of age. The results of TEM image analysis of mitochondrial size are shown in Fig. 5A. Mitochondria of R1441G HEM and HOM mice shrank and were smaller in size compared to *hLRRK2* mice.

We next investigated the effect of R144G1 on the expression levels of mitochondrial fission (i.e., Drp1 and Fis1) and fusion proteins (i.e., OPA1, Mfn1, and Mfn2) in brain tissue. Compared with WT *hLRRK2* mice, a significant increase in Drp1 (Fig. 5D, n = 3 per group) in R1441G HOM mice and Fis1 levels (Fig. 5E, n = 3 per group) was observed in R1441G HEM mice, while OPA1, Mfn1 and Mfn2 levels remained unchanged (data not shown). These findings suggest an increased level of mitochondrial fission and no change in the fusion process.

### The lysosome morphology was defective in SNc regions of Tg mice with *hLRRK2* R1441G by TEM

**Fig. 6:**
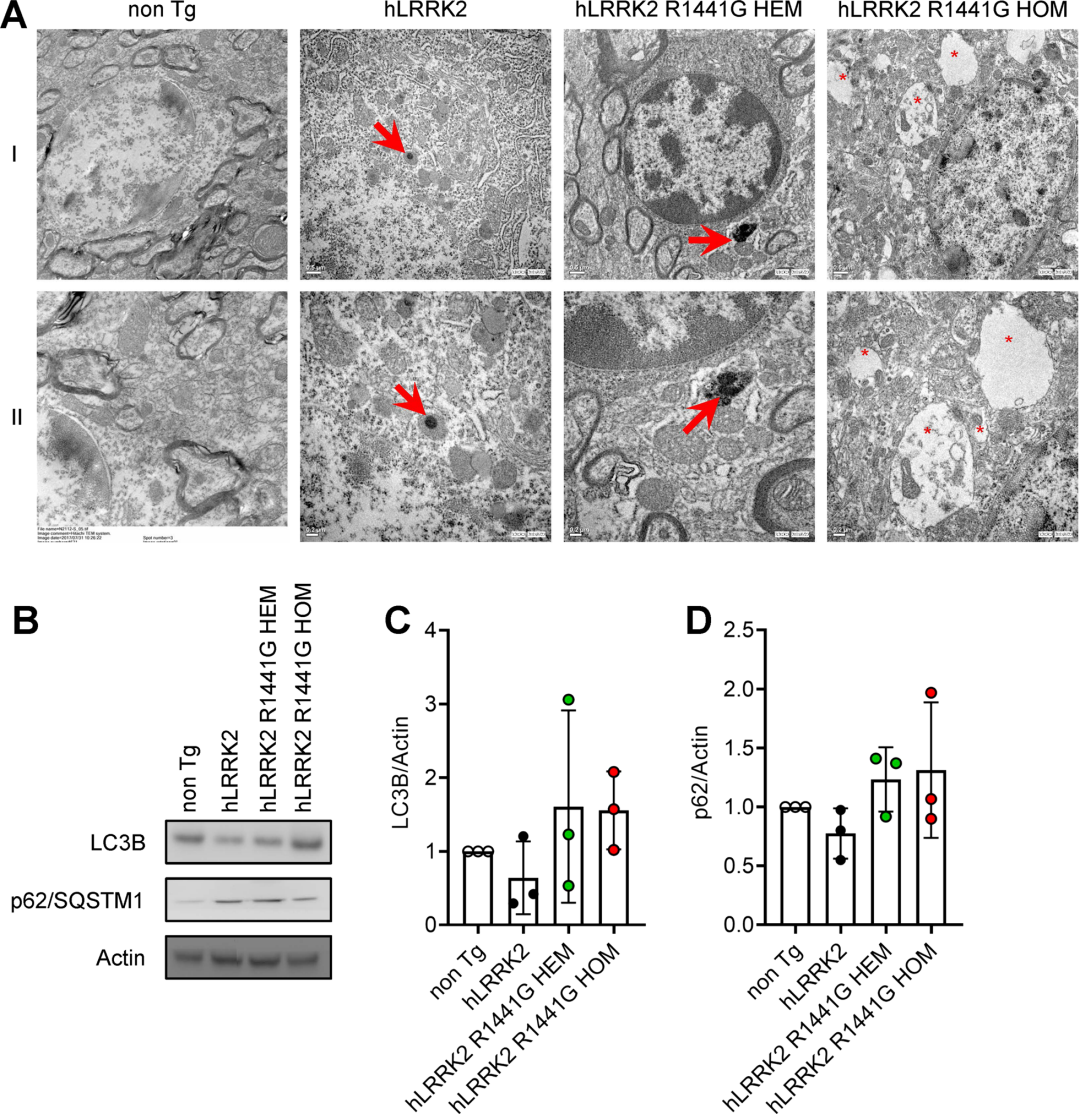
*LRRK2* R1441G HOM mice accumulated more autophagosomes in the SNc. **A** Transmission electron microscopic images of autophagosomes from the SN of 12-month-old *hLRRK2* and R1441G transgenic mice. Selected regions in images at different magnifications (I, 10,000×; II, 20,000×). Arrows indicate autolysosomes, and asterisks indicate autophagosomes. Markers of autophagy (LC3 and p62) in the SNc of *hLRRK2* and R1441G transgenic mice were determined by western blotting. p62 undergoes degradation at the early phase of autophagy. p62 in mitochondria serves as an adapter for autophagosome recognition. The data appear to downregulate the autophagy process, as observed by the increasing LC3-II conversion and the accumulation of p62, a marker of autophagic degradation

We further characterized the lysosome morphology in *hLRRK2* R1441G mutant Tg mice. TEM was used to observe the effect of the *hLRRK2* R1441G mutant on the lysosome morphology. In *hLRRK2* mice, lysosomes appeared as dense, spherical, membrane-enclosed vesicles (Fig. 6A). However, we observed abnormal lysosomes in R1441G HOM mice. First, we found enlarged membrane-bound vesicles that were filled with membranous and granular contents. In addition, we found the formation of closed autophagosomes fused with lysosomes.

To separately analyze the impact of LRRK2 R1441G on autophagic pathways, we first monitored the levels of microtubule-associated protein light chain 3 (LC3), a well-established marker for macroautophagy. Meanwhile, the levels of LC3 and p62/SQSTM1 weren’t statistically significant in mice expressing *hLRRK2* R1441G HEM and HOM (Fig. 6C and D).

## Discussion

Regarding the ideal control for a mutant mouse model overexpressing *LRRK2* Tg, a large proportion of previous PD studies in Tg mutant mice have used non-Tg mice as controls [7]. However, Tg mice are generated by inserting a foreign gene into the genome. When an exogenous gene is added to the mouse genome, it often leads to phenotypic changes. For example, bacterial artificial chromosome (BAC) *LRRK2* WT Tg mice were recently reported to show enhanced motor performance and striatal dopamine transmission compared to non-Tg mice [31]. Therefore, we believe that the ideal controls for mutant Tg mice are Tg animals expressing the WT allele at comparable levels to the mutant mice to control for the effects of overexpression by itself [10]. Accordingly, we used HEM *hLRRK2* WT Tg mice as the ideal controls and HEM and HOM *hLRRK2* R1441G Tg mice as comparison groups. This is the major difference between our study in the current Tg mouse model and previous studies in Tg mice overexpressing *hLRRK2* with the R1441G mutation.

In the present study, we found that *hLRRK2* mice showed hyperactivity and enhanced performance in motor function tests compared with non-Tg mice (Figs. 2 and 3). The results we find are consistent with previous results [31]. Compared with age-matched *hLRRK2* Tg mice, the *hLRRK2* R1441G HOM Tg mice expressed obvious age-dependent motor deficits at 12 months of age in our study (Figs. 2 and 3). Grossly, the mice did not show outward abnormalities or muscle wasting. In the OFT for the assessment of spontaneous movement and anxiety, the mice with HOM *hLRRK2* R1441G showed decreased ambulatory and fine activities. Moreover, this group of mice also revealed a reduction in time spent in the central and peripheral zones of the maze (Fig. 2E–G), supporting the coexistence of anxiety [48]. The motor dysfunction is consistent with the findings of *hLRRK2* R1441G Tg mice, which showed age-dependent motor disability evaluated by the administration of apomorphine and drug-induced rotational behavior [33]. Regarding rearing behavior in the OFT, there was a decreased frequency of rearing, indicating impaired vigilance and/or exploration [54]. The motor dysfunction of the *hLRRK2* R1441G Tg mice mimics the general bradykinesia of PD in humans. Impaired rearing behavior has been observed in mice with dopamine depletion in 6-hydroxydopamine mice [61]. In the gait analysis by the CatWalk system (Fig. 3), the decreased swing velocity, stride length, and cadence in 12 month-old *hR1441G* HOM mice were similar to the characteristics of the gait pattern, small steps, slow and shuffling gait-observed in the PD patients. The above findings in the present study demonstrated both motor and cognitive dysfunction in the *hLRRK2* R1441G Tg mouse, which resembles the motor and nonmotor symptoms in human PD [4].

By the current criteria, a neuropathologic diagnosis of sporadic PD requires both neuronal loss in the substantia nigra pars compacta (SNpc) and the presence of α-synuclein [43]. However, several reports of autopsy in cases with *LRRK2* G2019S or R1441G mutations linked to PD may challenge these criteria [1, 16, 18, 37]. Patients with a family history of parkinsonism and *LRRK2* G2019S mutation present with characteristic motor features, drug response and clinical course but no α-synuclein positive inclusions in the dopaminergic neurons and neurites in the substantia nigra [1]. Our data showed no difference in phospho-Ser129 α-synuclein in the substantia nigra of *LRRK2* R1441G Tg mice with or without the LRKK2 R1441G mutation by western blot. The immunobiochemical examination of TH-positive neurons in the substantia nigra did not show a significant reduction in the neuronal number between *hLRRK2* and *hLRRK2* R1441G Tg mice (Additional file 1: Fig. S1). However, the [^18^F]FDOPA PET study showed significantly decreased uptake of ligand in the striatum in both HEM and HOM *hLRRK2* R1441G Tg mice (Fig. 4E). This finding suggests that the dysfunction of nerve terminals in the nigrostriatal system occurs prior to dopamine neuron loss in this *hLRRK2* R1441G Tg mouse model, which is consistent with previous studies in the BAC Tg mouse model expressing the human disease-causing *LRRK2* (R1441G) mutant [32]. In this BAC Tg mouse model, axonal degeneration presented with spheroids in the medial forebrain bundle and striatum at 2–4 and 9 months. In other genetic or toxic models of parkinsonism such as adeno-associated virus (AAV) A53T or 1-methyl-4-phenyl-1,2,3,6-tetrahydropyridine (MPTP) mice, the results showed retrograde axonal degeneration before the change in dopamine neurons in the substantia nigra [39, 46]. Furthermore, there is also evidence in the human study of PD indicating that axonal degeneration is an early and predominant feature. The dopamine transporter (DAT) and vesicular monoamine transporter (VMAT) study showed 50–70% decreased uptake when the DA neuron loss was 30%. Taken together, the evidence suggests that axonal degeneration and synaptic changes occur are prior to DA neuron death in the present Tg mouse models and human PD study.

In this study, we investigated the changes in GTPase activity in both R1441G HEM and HOM mutation mice; we found that there was decreased GTPase activity in the HEM mutation but increased GTPase activity in the HOM mutation. This is consistent with previous in vitro studies that showed that the rate of GTP hydrolysis was reduced in cellular modes transfected with the HEM mutants of R1441C/G and Y1699C [23, 29, 32]. However, none of the previous studies investigated the effect of HOM mutation on GTPase activity. The present study is the first to demonstrate that *hLRRK2* R1441G HOM mice have higher GTP hydrolysis activity than *hLRRK2* mice (Fig. 1D). To explore the source of the increased GTPase activity, we confirmed that it was yielded from mitochondrial fission protein in the Tg mice. Our results showed increased Drp1 expression on mitochondrial fragments of R1441G HOM mice (Fig. 5). However, we did not find a change in mitochondrial fusion proteins (Opa-1, Mfn-1, and Mfn-2, Fig. 5A). These findings are compatible with the EM study, which revealed shrinkage and small size phenomena in SN mitochondria in R1441G HOM mice by TEM (Fig. 5A). In studies on murine primary neurons and human neuroblastoma, the interaction between endogenous LRRK2 and the fission regulator Drp1 increased Drp1 phosphorylation and mitochondrial fission [42, 60]. This LRRK2- and Drp1-dependent mitochondrial fragmentation is enhanced by overexpression of WT and R1441C *LRRK2* but can be reversed by inhibiting Drp1 or increasing fusion [52, 60]. Studies have shown that the phosphorylation of Drp1 at S616 causes fission. Notably, increased S616 phosphorylation has been observed in patients with sporadic PD [8, 47].

The autophagy–lysosomal pathway is another important mechanism for the pathophysiology of PD with *LRRK2*. According to our results, lysosomes are well resolved in *hLRRK2* mice. In contrast, lysosomes were enlarged and clustered in age-matched R1441G HOM mice (Fig. 6A). Meanwhile, the levels of LC3 and p62/SQSTM1 were not statistically significant (Fig. 6C and D). This finding isn’t consistent with a previous study showing an accumulation of autophagic vacuoles, with increased levels of p62 as a marker of autophagy in HEK-293 cells with mutations in the GTPase domain (e.g., R1441C) [3]. Furthermore, the autophagy–lysosomal pathway is also impaired in the absence of LRRK2, involving lipofuscin granule accumulation and altered levels of LC3-II and p62 [57]. An investigation of the regulation of the tissue specificity of LRRK2 expression by autophagy showed the age-dependent accumulation of autophagic vacuoles in the cortex and striatum of R1441C and G2019S Tg mice, suggesting that LRRK2 expression is regulated by autophagy specifically in neuronal somas and axial processes from the cortex and striatum [44]. Regarding the finding of obvious change in lysosomal morphology were seen in the SNc of HOM *hLRRK2* R1441G mice by TEM, it is notable that there was no statistically significant difference in autophagy markers in the present study. However, to make it clear, future studies are precious by increasing the number of studied animals which could either reduce the standard errors of mean value or alleviate the variation of the autophagy process at different stages in HOM *hLRRK2* R1441G Tg mice.

LRRK2 is constitutively phosphorylated at Ser935, which responds to LRRK2 kinase inhibition [11]. Ser935 phosphorylation is decreased by the PD-linked mutations R1441C and Y1699C [40], while these pathogenic variants show increased kinase activity toward Rab GTPases [51]. An intriguing aspect of our study was the complete loss of LRRK2 phosphorylation at Ser935 in the SNc of HOM *hLRRK2* R1441G mice, but not in HEM *hLRRK2* R1441G Tg mice (Fig. 1E). The mechanism underlying this finding is uncertain. One of the speculations is that the inserted genetic material might cause a mutation of the gene regulating Ser935 phosphorylation in an autosomal recessive pattern [10, 20]. Thus, the effect would not be found in HEM *hLRRK2* R1441G Tg mice but would be expressed in HOM *hLRRK2* R1441G Tg mice. Complete loss of the Ser935 phosphorylation site has been described by a previous study in the *LRRK2* Ser910Ala/Ser935Ala double knockin mice [65]. In this double knockin mouse line, no anxiety or motor dysfunction obverted at 9 months of age. Therefore, we surmised that the abnormal phenotype of the HOM *hLRRK2* R1441G Tg mice in our study might not be caused by the loss of Ser935 phosphorylation. This phosphorylation site did not have a significant effect on the total protein level of LRRK2 in the different strains of Tg mice (Fig. 1B).

There are several limitations in the present study. First, the BAC Tg mice inserted with HEM *hLRRK2* WT (FVB/N-Tg(*LRRK2*)1Cjli/J, no. 009610) and HEM *hLRRK2* R1441G (FVB/N-Tg(LRRK2*R1441G)135Cjli/J, no. 009604) genomes [32] were used in this study. BACs have been used to some degree of success with mice while studying neurological diseases such as PD [63]. BACs are preferred for these kinds of genetic studies because they accommodate much larger sequences without the risk of rearrangement and are therefore more stable than other types of cloning vectors [5]. However, the enhancement of expression may result in the random integration of multiple copies of the transgene [63]. One of the most frequently described issues in BAC transgenesis consists of unwanted effects caused by integration of the large transgene in another gene. For example, this scenario can lead to a functional knockout of the affected gene or cause a change in its expression pattern [63]. This may lead to misinterpretation regarding the effect of transgene expression. A recent study in HEM *hLRRK2* R1441G Tg mice (FVB/N-Tg(LRRK2*R1441G)135Cjli/J) showed that *hLRRK2* R1441G integrated into mouse chromosome 1, causing a 436 bp deletion in an intron of the Khdrbs2 (KH domain containing, RNA binding, signal transduction associated 2) gene [20]. However, the deletion in introns did not affect protein sequences. Therefore, we propose that the risk of transgene inserted into the random genomic locus in the homogenous overexpression Tg mice is very low in this study. Second, we did not use GTPase inhibitors as a pharmacological treatment for motor dysfunction in these Tg mice [22]. Meanwhile, the changes in kinase activity in association with the increased GTPase activity in the SN were not measured in this study. Finally, whether the loss of Ser935 phosphorylation can cause the delocalization of LRRK2 in the soma and axon in this animal model also requires further elucidation.

## Conclusions

We observed the alternations in the phenotype, functional image and morphological changes of the mitochondria and autophagosomes between the ideal control group (*hLRRK2*) and HOM *hLRRK2* R1441G (Figs. 1, 2, 3, 4, 5 and 6). There were also several differences in the manifestations between the non-Tg and HOM *hLRRK2* R1441G groups, including body weight, anxiety behavior, motor dysfunction and GTPase activity. Taken together, the evidence suggests that HOM *hLRRK2* R1441G overexpressing Tg mice are a novel genetic model of PD that could be used for the study of the pathophysiology and new targets of pharmacological therapy. However, given the limitation of the data presented in this study, it needs further studies to elucidate the usefulness of HOM *hLRRK2* mice for pre-clinical modeling of PD.

## Supplementary Information

**Additional file 1: Figure S1.** Immunohistochemical staining of TH-positive neurons in the midbrain of adult *LRRK2* R1441G (12 months old) did not show a significant difference in the positive neurons between groups with or without genetic mutation.

## Data Availability

All data generated or analyzed during this study are included in this published article and its additional files.

## Abbreviations

AAV: Adeno-associated virus
apoB: Apolipoprotein B
BAC: Bacterial artificial chromosome
BOS: Base of support
CB: Cerebellum
DAT: Dopamine transporter
FOV: Field of view
GTPase: Guanosine triphosphatase
HEM: Hemizygous
hLRRK2: Human LRRK2
HOM: Homozygous
LRRK2: Leucine-rich repeat kinase 2
MPTP: 1-Methyl-4-phenyl-1,2,3,6-tetrahydropyridine
non-Tg: Non-transgenic
OFT: Open field test
PD: Parkinson’s disease
PFA: Paraformaldehyde
Pi: Inorganic phosphate
S: Striatum
SNc: Substantia nigra pars compacta
SUV: Standardized uptake value
SUVR: SUV ratio
TEM: Translate electron microscope
Tg: Transgenic
VMAT: Vesicular monoamine transporter
VOI: Volumes of interest
WB: Western blotting
WT: Wild-type
