# Supplementary figures and images for: Homozygous mutation of the LRRK2 ROC domain as a novel genetic model of parkinsonism
Source: J Biomed Sci. 2022 Aug 14;29:60. doi: 10.1186/s12929-022-00844-9 (PMC9375908; doi:10.1186/s12929-022-00844-9)

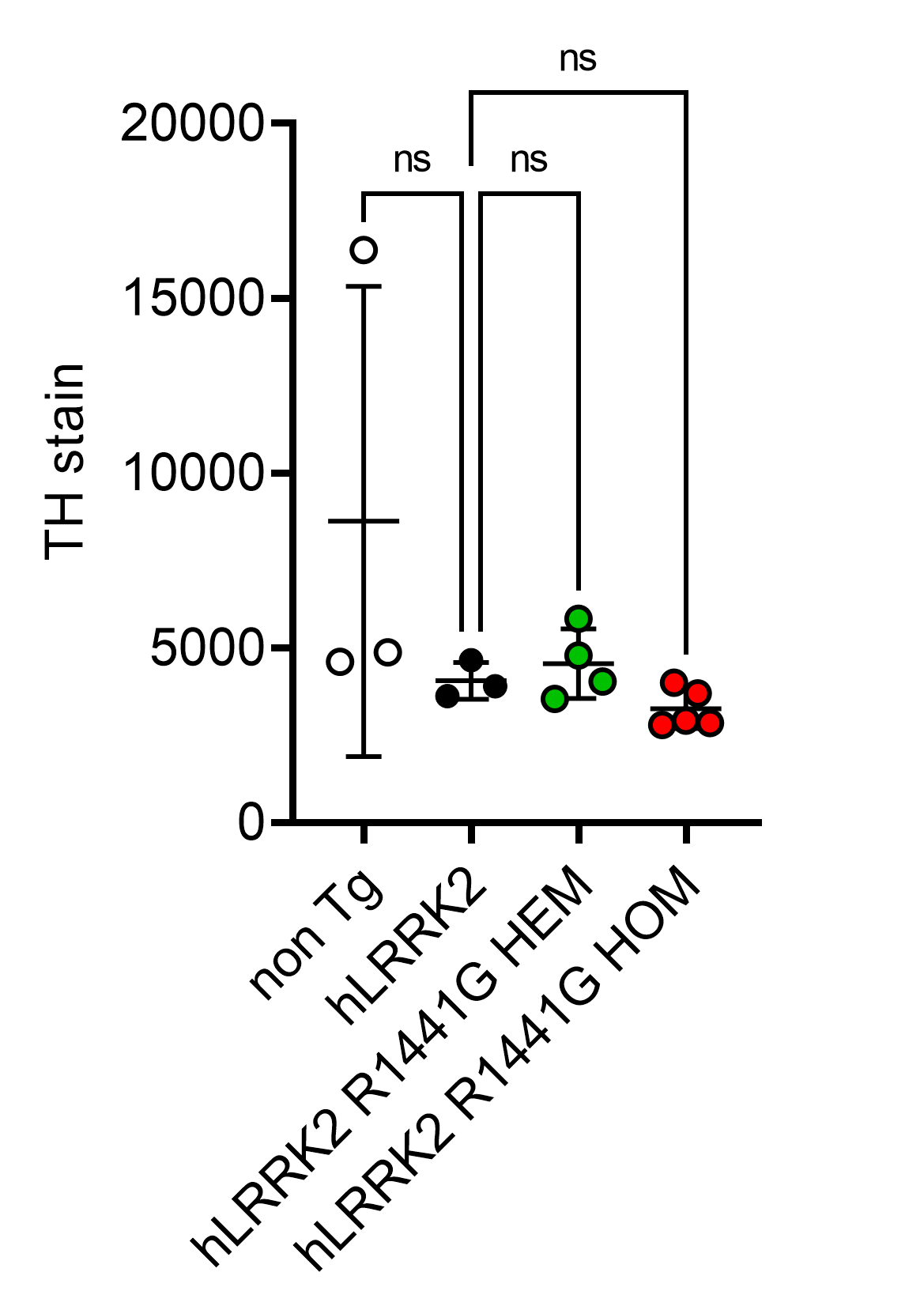

Supplement: Supplementary file 1 — Additional file 1: Figure S1. Immunohistochemical staining of TH-positive neurons in the midbrain of adult LRRK2 R1441G (12 months old) did not show a significant difference in the positive neurons between groups with or without genetic mutation. [file 12929_2022_844_MOESM1_ESM.tif]
